# 

**Published:** 1895-04-13

**Authors:** 


### CONTENTS.

Cambridge Shows Oxford the Way - - - - - - - 19

The Summer Session - - - - - - - - - - 20

Drink and Accidents - - - - - - - - - - 20

On Modern Progress in Ophthalmic Medicine and Surgery. By R. Brudenell Carter, F.R.S. - - - - 21

The Panacea in English Medicine. IV.—Mithridatium - 22

Medical Progress and Hospital Clinics:

The Prevention of Puerperal Fever - - - - - - - 25

Club Foot.—II. - - - - - - - - - - - - 26

Progress in Medicine.—Diseases of the Circulatory System - 23

Progress in Surgery.—Genito-Urinary Surgery - - - - 29

Therapeutic Notes.—Papain - - - - - - - - - 30

Annotations.—Hereditary Disqualification for Marriage—Adulterated Milk at Public Institutions—A Patent for Antitoxin - - 23

Notes and News - - - - - - - - - - - - - 24

The Institutional Workshop:

Within the Hospitals.—Samaritan Free Hospital for Women and Children - - - - - - - - - - - - - 31

Hospital Construction.—Hospital for Thurso District - - 32

Practical Departments.—Heating and Ventilation - - - 33

Hospital Festivals and Meetings - - - - - - - 33

Construction Notes - - - - - - - - - - - 34

An American Hospital—Scraps and Gleanings - - - 35

The Book World of Medicine and Science - - - - 35

### EXTRA SUPPLEMENT.—THE NURSING MIRROR.

News from the Nursing World.—With the Sick Children—Westminster Hospital Bazaar—Help Sorely Needed—District Nursing at Haggerston—The Last Thing in Badges—Bazaar at Derby—Promiscuous Charities—"A Young Person" as "Nurse" Kilmarnock—Who Attends Health Lectures?—Sympathetic or Antagonistic?—A Trained Nurse—Holland—Short Items - - ix

Elementary Anatomy and Surgery for Nurses. By W. McAdam Eccles, M.B., M.S., F.R.C.S. - - - - - - xi

Invalids at Netley - - - - - - - - - - - - xii

District Nursing at Worthing—Where to Go - - - - xii

A Great Movement: The Nurses' Co-operation. III. - xiii

Addenbrooke's Hospital, Cambridge - - - - - - xiv

Burdett's Official Nursing Directory - - - - - - xiv

Notes and Queries - - - - - - - - - - - xiv

Dress and Uniform.—St. Thomas's Hospital - - - - xv

Everybody's Opinion—Our American Letter - - - - xvi

